# Regional thalamic neuropathology in patients with hippocampal sclerosis and epilepsy: A postmortem study

**DOI:** 10.1111/epi.12403

**Published:** 2013-10-18

**Authors:** Barah Sinjab, Lillian Martinian, Sanjay M Sisodiya, Maria Thom

**Affiliations:** * Department of Clinical and Experimental Epilepsy, Institute of Neurology and National Hospital for Neurology and NeurosurgeryLondon, United Kingdom; † Divisions of Clinical Neurology, Institute of Neurology and National Hospital for Neurology and NeurosurgeryLondon, United Kingdom; ‡ Epilepsy Society, Chalfont St. PeterBucks, United Kingdom; § Divisions of Neuropathology, Institute of Neurology and National Hospital for Neurology and NeurosurgeryLondon, United Kingdom

**Keywords:** Thalamus, Gliosis, Mediodorsal nucleus, Hippocampal sclerosis

## Abstract

**Purpose:**

Clinical, experimental, and neuroimaging data all indicate that the thalamus is involved in the network of changes associated with temporal lobe epilepsy (TLE), particularly in association with hippocampal sclerosis (HS), with potential roles in seizure initiation and propagation. Pathologic changes in the thalamus may be a result of an initial insult, ongoing seizures, or retrograde degeneration through reciprocal connections between thalamic and limbic regions. Our aim was to carry out a neuropathologic analysis of the thalamus in a postmortem (PM) epilepsy series, to assess the distribution, severity, and nature of pathologic changes and its association with HS.

**Methods:**

Twenty-four epilepsy PM cases (age range 25–87 years) and eight controls (age range 38–85 years) were studied. HS was classified as unilateral (UHS, 11 cases), bilateral (BHS, 4 cases) or absent (No-HS, 9 cases). Samples from the left and right sides of the thalamus were stained with cresyl violet (CV), and for glial firbillary acidic protein (GFAP) and synaptophysin. Using image analysis, neuronal densities (NDs) or field fraction staining values (GFAP, synaptophysin) were measured in four thalamic nuclei: anteroventral nucleus (AV), lateral dorsal nucleus (LD), mediodorsal nucleus (MD), and ventrolateral nucleus (VL). The results were compared within and between cases.

**Key Findings:**

The severity, nature, and distribution of thalamic pathology varied between cases. A pattern that emerged was a preferential involvement of the MD in UHS cases with a reduction in mean ND ipsilateral to the side of HS (p = 0.05). In UHS cases, greater field fraction values for GFAP and lower values for synaptophysin and ND were seen in the majority of cases in the MD ipsilateral to the side of sclerosis compared to other thalamic nuclei. In addition, differences in the mean ND between classical HS, atypical HS, and No-HS cases were noted in the ipsilateral MD (p < 0.05), with lower values observed in HS.

**Significance:**

Our study demonstrates that stereotypical pathologic changes, as seen in HS, are not clearly defined in the thalamus. This may be partly explained by the heterogeneity of our PM study group. With quantitation, there is some evidence for preferential involvement of the MD, suggesting a potential role in TLE, which requires further investigation.

Sclerosis of the hippocampus is one of the most common and best characterized pathologies identified at both postmortem (Margerison & Corsellis, 1966; Meencke et al., 1996) and in surgical series of patients with epilepsy, and is particularly associated with the syndrome of mesial temporal lobe epilepsy (MTLE; Blumcke, 2009). In surgical series, hippocampal sclerosis (HS) is typically encountered in specimens from young adults, in the context of refractory seizures, with sclerosis visible on magnetic resonance imaging (MRI) and confirmed in resected specimens (Wieser, 2004). Neuropathology studies have, from the outset, recognized that more extensive, albeit more subtle, pathology may accompany HS (Cavanagh & Meyer, 1956). In recent years, clinical and neuroimaging studies have also moved away from a “hippocampocentric” view of MTLE (Thom et al., 2010a; Engel & Thompson, 2012). The additional structures altered include those anatomically linked to, or in proximity of, the hippocampus, such as the amygdala (Bernasconi et al., 2003), entorhinal cortex (Bernasconi et al., 1999; Jutila et al., 2001; Bernasconi et al., 2003; Bonilha et al., 2004; Keller et al., 2004), temporal pole (Coste et al., 2002; Sankar et al., 2008), and cingulate gyrus (Bernhardt et al., 2008; Bonilha et al., 2010a). This wider network of disease may correlate with an extended zone of epileptogenesis and explain poor outcomes in some patients following focal surgery, as well as offer an explanation for comorbidities associated with HS as progressive memory decline.

Neuropathologic study of the thalamus is merited in patients with HS for several reasons. The hippocampus has important reciprocal connections to the thalamus (Hirai & Jones, 1989; Herrero et al., 2002). The main output pathway of the hippocampus (fornix) projects through the medial mamillary nucleus to the anteroventral thalamic nucleus (AV); the anterior and lateral dorsal thalamic nuclei are both reciprocally connected with the limbic cortex; the mediodorsal nucleus (MD) also receives input from the amygdala, entorhinal cortex, and temporal pole (Nieuwenhuys et al., 2008a). A number of experimental studies have indicated that the thalamus plays a crucial role in seizure initiation and modulation, thereby strongly suggesting that it may be an important part of the substrate for MTLE (Bertram et al., 2008; Sloan & Bertram, 2009; Sloan et al., 2011). In addition, there are data from electroclinical studies supporting synchronization of activity in the thalamus in association with temporal lobe seizures (Guye et al., 2006). The neuroimaging literature also points toward volume loss occurring in the thalamus in TLE, although there are inconsistencies between studies regarding its association with HS, whether greater volume loss is observed ipsilateral to side of HS, as well as the regional distribution of pathology within the thalamus (DeCarli et al., 1998; Dreifuss et al., 2001; Behrens et al., 2003; Natsume et al., 2003; Bonilha et al., 2004; Labate et al., 2008; Kim et al., 2010; Mueller et al., 2010; Alhusaini et al., 2012).

Detailed histologic studies of the thalamus in TLE are lacking. Neuronal loss has been demonstrated in experimental models (Bertram & Scott, 2000; Bertram et al., 2001). In a single postmortem study from 1966, thalamic damage was frequent in a series of patients with TLE and HS (Margerison & Corsellis, 1966), but not further detailed. Our aim was to investigate the extent and regional distribution of thalamic pathology in a postmortem epilepsy series using quantitative immunohistochemistry.

## METHODS

### Case and tissue selection

Postmortem cases were selected from the archives of the Division of Neuropathology at the National Hospital for Neurology and Neurosurgery, Queen Square, London. The local ethics committee of the National Hospital for Neurology and Neurosurgery and the Institute of Neurology has given approval for neuropathologic epilepsy studies, and era-appropriate consent was obtained from patients' next-of-kin.

Twenty-four epilepsy cases (age range 25–87; mean age 56.5 years) were selected together with eight controls (age range 38–85; mean age 59.8 years) without significant neurologic disease or epilepsy (Table S1). The majority of patients had lifelong, drug-resistant epilepsy. Details of epilepsy history, seizure type, and epilepsy syndrome as well as recent seizure control are recorded in Table S1. The patients for this study were selected according to the presence (or not) of neuropathologically confirmed HS. The diagnosis of HS was based on previous criteria (Thom et al., 2005, 2009), and the pattern was defined as classical HS (CHS: neuronal loss and gliosis in CA1 and CA4) or atypical HS either CA1 predominant sclerosis (CA1p: neuronal loss in CA1) or end folium sclerosis (EFS: neuronal loss in CA4) (Thom et al., 2010b); this equates with HS International League Against Epilepsy (ILAE) types 1, 2, and 3, respectively, in the new classification (Blumcke et al., 2013). Cases where only end floium gliosis (EFG) was present but with no neuronal loss were considered as No-HS. HS was classified as unilateral HS (UHS; 11 cases), bilateral HS (BHS; 4 cases) or No-HS (9 cases) (see Table S1).

The coronal level represented in the sections of thalamus varied from the subthalamic nucleus to the lateral dorsal thalamic nucleus. In 18 cases thalamic nuclei were sampled at the time of the postmortem examination, and formalin-fixed paraffin-embedded, paired tissue blocks from both left and right sides of thalamus at the same coronal level were available. In six cases, paired tissue blocks of left and right thalamus were sampled from the remaining formalin-fixed brain tissue. In some cases more than one coronal level was included through the thalamus to maximize the sampling of the subnuclei. In the six cases that were re-sampled, fixation times varied from 3 to 15 years (mean 8.6 years) compared to 2 to 8 weeks fixation times in the other 18 epilepsy cases. In one case (EP295-UHS), only one brain hemisphere was available for examination. We excluded epilepsy cases with second pathologies involving the thalamus although evidence of old trauma or cerebrovascular injury elsewhere in the brain was present in some cases (Table 1); all the patients in the series had low Braak stage for Alzheimer's disease. Controls were selected from our own archives and from the Queen Square Brain Bank. For all control cases bar one, only one hemisphere (and thalamus) was available for examination. In all cases a reference hematoxylin and eosin (H&E) and/or LFB/CV (Luxol fast blue/cresyl violet)–stained section was available for confirmation and delineation of the anatomical boundaries of thalamic subnuclei (Hirai & Jones, 1989).

**Table 1:** Results of neuropathology quantitative analysis in thalamic subnuclei in epilepsy groups and controls

Group	Side	GFAP% [SD] N = number of thalamus sections				Synaptophysin% [SD] N = number of thalamus sections				Cresyl violet (Neuronal density × 10^−5^/μm^2^) [SD] N = number of thalamus sections			
		VL	MD	AV	LD	VL	MD	AV	LD	VL	MD	AV	LD
Epilepsy cases	Unilateral HS (n = 11)													
	HS-side	16.9 [7]	18.4 [8]	16.2 [9.5]	13.6 [4]	38 [11]	35 [12]	62	33 [15]	7.4 [6.3]	7.8 [2.4]	7.4 [2.4]	14 [13.9]
		n = 11	n = 10	n = 4	n = 4	n = 5	n = 4	n = 1	n = 3	n = 11	n = 10	n = 4	n = 4
	No-HS-side	16.9 [7.6]	20.1 [10]	15.5 [9]	17.4 [7]	39.7 [35]	48 [33]	51.4 [15]	26.6 [21]	5.5 [1.8]	11.7 [6.1]	7.2 [2.6]	7.5 [1.7]
		n = 10	n = 9	n = 4	n = 3	n = 5	n = 4	n = 2	n = 2	n = 11	n = 9	n = 4	n = 3
	Bilateral HS (n = 4)	25 [17]	26 [19]	29.7 [16]	25.7 [26]	42.9 [9]	45.7	15		8.5 [14]	7.2 [3]	8.2 [1]	2.5 [0.9]
		n = 8	n = 6	n = 4	n = 3	n = 2	n = 1	n = 1		n = 8	n = 6	n = 4	n = 3
	No-HS (n = 9)	22.3 [10]	24 [10]	21.9 [14]	26.4 [16]	40.9 [6]	40.5 [6]	45.4 [12]	44.2 [5]	4.3 [1]	7.7 [2]	8.1 [2]	6.3 [1.9]
		n = 17	n = 16	n = 9	n = 7	n = 15	n = 14	n = 9	n = 6	n = 17	n = 16	n = 9	n = 7
Controls	Controls (n = 8)	27.3 [11]	26.2 [9]	24.3 [19.4]	30.8 [9]	34 [8]	30 [8]	55 [13]	41 [13]	3.6 [1]	5.6 [1.9]	2.8 [1.9]	4.4 [2.1]
		n = 9	n = 9	n = 2	n = 7	n = 9	n = 9	n = 2	n = 7	n = 9	n = 9	n = 2	n = 7
Sig*									p < 0.01	p < 0.05		p < 0.05	

The GFAP and synaptophysin are shown as mean values of the overall percentage of positive staining and the cresyl violet as neuronal density per unit area (standard deviation in brackets).

HS, hippocampal sclerosis; VL, ventrolateral nucleus; MD, mediodorsal nucleus; AV, anteroventral nucleus; LD, lateral dorsal nucleus.

* Significant differences between the four groups (including values from both center and right hemispheres) are shown using the Kruskal-Wallis test.

### Tissue preparation

For each case, 7-μm–thick formalin fixed paraffin embedded sections from the left and right side of thalamus were dewaxed, rehydrated through graded alcohols and immersed in distilled water. CV stain was carried out on one section. Immunohistochemistry for glial fibrillary acidic protein (GFAP) and synaptophysin were carried out as follows. Endogenous peroxidase was quenched with 3% hydrogen peroxide in water. After relevant antigen treatments, sections were stained for 1 hour at room temperature with the following antibodies: polyclonal GFAP (1:1,500; Dako, Glostrup, Denmark) with proteinase K for enzyme digestion, synaptophysin (1:100, Dako) with ethylenediaminetetraacetic acid (EDTA) heat-mediated epitope retrieval. In cases with longer fixation times, synaptophysin staining was not included due to the known diminished labeling over time with this antibody (Liu et al., 2010). Labeling was detected with horseradish peroxidase kit (Dako Envision) and diaminobenzidine (DAB) for visualization. Sections were counterstained with hematoxylin.

### Quantitative analysis of stained sections

**Figure 1:**
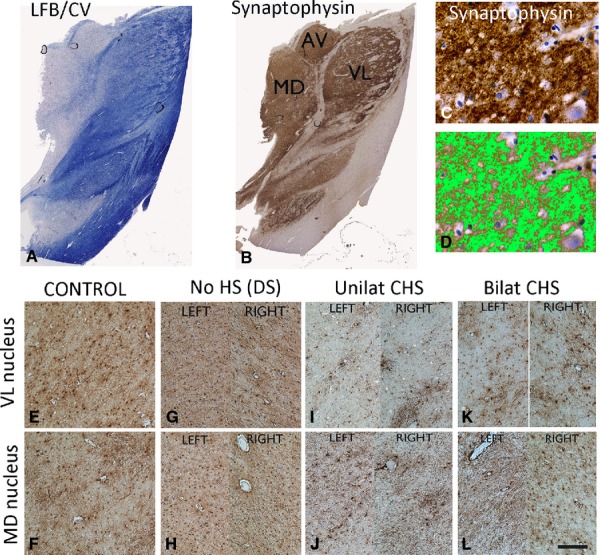
(A) Luxol/fast blue preparation from the thalamus at the level of the subthalamic nucleus highlighted the white matter pathways including the internal medullary laminae and (B) synaptophysin-stained preparation of the same case with three of the four subnuclei quantified indicated (MD, mediodorsal nucleus; VL, ventrolateral; AV, anteroventral). (C) Synaptophysin labeling within the VL at ×40 objective and (D) the same image with a green overlay used to detect the field fraction of immunostaining with the image analyzer. (E, F) In one of the controls with GFAP staining, an even distribution of positively labeled astrocytes and processes were observed in the VL (E) and MD (F). Similarly in a patient with No-HS and Dravet syndrome there was no qualitative variability between GFAP staining in the VL (G) and MD (H) or between the left and right sides (as labeled), which was confirmed with quantitative values. In a patient with TLE and unlilateral left-sided CHS shown in (I, J) there was no difference between the level of staining in the left or right VL subnuclei (values of 12.5% and 11% field fraction staining at ×40). Higher field fractions were noted in the MD (J) but were not different between the left (16%) and right sides (17.4%). (K, L) In a patient with bilateral CHS, there appeared slightly greater, but symmetrical increase in GFAP in the VL compared to the unilateral CHS (13.1% and 14.7%, left and right, respectively) in addition to a symmetrically greater increase in gliosis in the MD (19.9% and 19.8%, left and right, respectively). Bar = 100 μ for (E–L).

A commercial image analysis system (Histometrix; Kinetic Imaging, Liverpool, United Kingdom) with a Zeiss Axioskop (Cambridge, United Kingdom) microscope was used for the field fraction analysis and for estimation of neuronal number. On the GFAP, synaptophysin, and CV sections, regions of interest (ROIs) were outlined with reference to the LFB/CV–stained section for delineation of the thalamic subnuclei based on the regional cytoarchitecture (Hirai & Jones, 1989; Nieuwenhuys et al., 2008b). Four different thalamic nuclei were selected for inclusion, because of their known connections with the hippocampus, previous literature implicating a role in epilepsy, or the efficacy of their identification in tissue sections. They included the anteroventral nucleus (AV), lateral dorsal nucleus (LD), mediodorsal nucleus (MD) and the ventrolateral nucleus (VL). The entire area of each nucleus was outlined on the image analyzer program at ×2.5 magnification (Fig. 1). We aimed to include the maximal anatomic extent of each subnucleus, but with care to exclude peripheral myelinated fiber tracts, such as the internal and external medullary lamina. We did not further subdivide the nuclei into their components, for example, the magnocellular, parvocellular, and paralaminar divisions of the MD, but included the entire area available of each nucleus.

Field fraction analysis was carried out for GFAP-and synaptophysin-immunostained sections (Blanc et al., 2011) by calculating the percentage of pixels stained in a given field at ×40. The RGB (red-green-blue) detection threshold was set for the first field to detect the majority of immunostaining, but minimizing nonspecific detection (Fig. 1C,D). Uniform random sampling of fields examined at high magnification within each ROI was then carried out. In a pilot study, a sampling fraction of 10% for GFAP, 15% for synaptophysin for the VL and MD nuclei, and 100% sampling for the AV and LD nuclei was found to give reproducible measurements upon resampling, with and an average coefficient of error <0.1. Light intensity and the image analysis RGB detection thresholds were kept constant throughout the entire experiment for each case. Estimates of the percentage of GFAP-positive staining averaged over all fields for each of the four nuclei were calculated. For estimates of neuronal density on CV-stained sections, similar ROI were drawn and all cells with a neuronal morphology were counted at ×40; a sampling fraction of 15% was selected for the VL and MD and 100% in the LD and AV for optimal reproducibility of measurements.

Statistical analysis was carried out using SPSS (IBM) version 16 and the Mann-Whitney test, Kruskal-Wallis, and nonparametric correlations (Spearman's or Kendall's tests) for comparison of data between patient groups (p < 0.05 taken as significant).

## RESULTS

### Regional thalamic subnuclear pathology

We identified the MD and VL (subnuclei) in 92% of hemispheres and the AV and LD in 42%; in all cases except two, we were able to define at least three thalamic subnuclei per section (Fig. 1A). The thalamic structures showed a variable, patchy gliosis throughout the thalamus, more often in a perivascular distribution (Fig. 1G–I). In no epilepsy cases, with CV, synaptophysin, or GFAP did we identify distinct atrophy or sclerosis of a nucleus comparable to the degree of hippocampal damage in CHS. Quantitative analysis of all cases and controls, in each of the four subnuclei, did not show a significant correlation between GFAP, neuronal density, and synaptophysin measurements (Fig. 1C,D), apart from the LD, which showed a positive correlation between CV and synaptophysin (p < 0.05). In the control group, regarding regional variation in synaptophysin staining, higher mean field fractions were present in the AV than the MD (Table 1, p < 0.001) supporting a normal variation in synaptic density; there was, however, no significant difference in the variation of mean neuronal density on CV stain or astrocytic gliosis between subnuclei in controls. Comparison of all pathology measures between the epilepsy groups (UHS, BHS, and No-HS) and controls showed significant differences for neuronal density in the VL, MD, and LD (p < 0.01, p < 0.05 and p < 0.05; Table 1), with higher values noted in the patients with epilepsy.

### Comparison of left and right thalamic nuclei in unilateral HS group

We analyzed the patterns within patient subgroups. In the 11 UHS cases, there was a trend for significant reduction in the neuronal density in the MD ipsilateral to the side of HS compared to the contralateral side (p = 0.05). No significant differences were noted in other subnuclei, either with GFAP or synaptophysin staining. Furthermore, no significant differences were noted for similar comparisons in the BHS and No-HS epilepsy groups.

**Figure 2:**
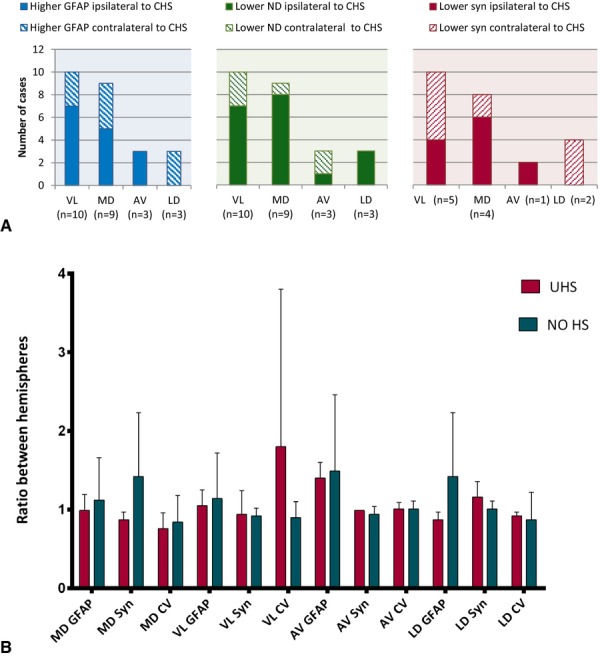
Comparison between left and right hemispheres in unilateral HS group. (A). The value for each measurement in each case in the thalamic subnuclei on the side ipsilateral to the side of CHS was compared to the contralateral side and value ranked as either higher or lower. The DM showed lateralization to the side of sclerosis in the majority of cases with all markers (B) Ratio of interhemispheric values between nuclei in epilepsy groups with unilateral hippocampal sclerosis (UHS) or without sclerosis (No-HS). In UHS cases this ratio is expressed as side ipsilateral to HS: contralateral side; in No-HS the ratio is left: right side. A ratio near to 1 implies little difference between the left and the right hemisphere. The mean values of the ratios in each group and the standard deviations are shown as bars. Although the lowest values for CV and synaptophysin were seen in the MD in UHS group, there were no statistically significant differences overall between UHS and No-HS groups. Furthermore, in the UHS cases there was no significant difference in ratios in regard to laterality of the sclerosis. VL, ventrolateral thalamic nucleus; MD, mediodorsal nucleus; AV, anteroventral nucleus; LD, lateral dorsal nucleus; syn, synaptophysin; CV, cresyl violet; ND, neuronal density; CHS, classical hippocampal sclerosis.

Direct comparison of measurement values between left and right sides within individual cases avoids any variability of staining intensity due to differences in tissue-fixation times and processing. Although no consistent patterns were seen, in UHS cases, greater values with GFAP and lower values with synaptophysin and neuronal density were seen for the majority of cases in the MD ipsilateral to the side of sclerosis (Fig. 2A) compared to other thalamic nuclei, suggesting more frequent pathology (neuronal loss, synaptic reduction, or gliosis) in this subnucleus. Furthermore, by expressing the measurements in each subnucleus on the sclerotic side as a ratio of the contralateral side, the lowest values for CV and synaptophysin (reflecting the greatest pathologic changes) were confirmed for the MD (Fig. 2B). However, when a similar analysis was carried out for the nine No-HS epilepsy cases, compared with the UHS cases, there was no statistically significant difference in these ratios (Fig. 2B). There was also no significant difference in these ratios in the UHS group regardless of whether the sclerosis was on the left or right side.

### Comparison of thalamic nuclei in relation to pattern of HS

Comparing CHS (ILAE type 1), atypical HS patterns (ILAE types 2 and 3), and No-HS epilepsy patients, lower values for synaptophysin labeling were noted ipsilateral to CHS in all thalamic nuclei, although these differences were not significant. There was a statistically significant difference between the mean ND on CV in the VL between these groups (p < 0.05) and a trend for a difference in GFAP in the MD (p = 0.08); both values were lowest in atypical HS.

### Comparison in relation to clinical and pathology feature

Clinicopathologic correlations showed an association of reduced synaptophysin labeling in the VL (subnucleus) in patients with TLE (p < 0.05) and lower neuronal density on CV in the AV in both TLE and partial epilepsy syndromes compared to other epilepsy cases (p < 0.01). Because most patients had generalized seizures in addition to partial seizures in this small group, it was not possible to assess thalamic damage in relation to seizure type. There was a negative correlation between the age of death and synaptophysin field fractions in the AV (p = 0.018). The mean age of onset of epilepsy was 8 years (range 0.3–28 years) and the mean duration of epilepsy (age of onset of habitual seizures until most recent seizure) was 45 years (range 2–86 years); there was a positive correlation between duration of epilepsy and the field fraction of GFAP in the VL (p < 0.05) and the AV (p < 0.05). There were no significant differences in any of the pathology measures in relation to whether the patient had refractory epilepsy or was in remission at the time of death. There were no significant correlations between the presence of cognitive decline, brain weight, the presence of traumatic brain injury, or cerebrovascular disease (CVD) and any of the measures in the thalamic nuclei. There were no significant differences in GFAP measures and CV neuronal density between cases that had short or long fixations. The synaptophysin was not assessed in long fixation cases, so statistical analysis was not carried out.

## DISCUSSION

There is converging clinical, experimental, and neuroimaging evidence that hippocampal sclerosis in epilepsy may represent part of a wider network of pathologic changes, including within the thalamus. In this first detailed neuropathologic study of the thalamus in epilepsy, we have demonstrated that, in contrast to the stereotypical patterns of sclerosis (neuronal loss and gliosis) observed in the hippocampus, cellular alterations within subthalamic nuclei are milder, less clearly defined, varying both in regional distribution, nature, as well as severity. It remains to be established whether any of the extended regional pathologies accompanying HS are as a result of the same initial “insult,” represent secondary progressive changes, such as retrograde/anterograde degeneration along known projections, or arise independently as a result of the seizures. Indeed all of these mechanisms may operate. We employed three standard neuropathologic measurements in this initial assessment of the thalamus to quantify total synaptic density as a measure of connectivity (synaptophysin), neuronal number (cresyl violet), and gliosis (GFAP) in selected subnuclei. These measurements did not correlate with each other (except for the LD nucleus), suggesting that the pathologic processes of astrocytic gliosis and alterations in synaptic and neuronal density may not occur synchronously.

We did not demonstrate significant differences between all the patients with epilepsy compared to controls. Indeed for some subnuclei, higher neuronal densities were noted in epilepsy groups than in controls. A possible explanation for this is that gliosis (and therefore tissue volume reduction) may predominate over neuronal loss, resulting in a paradoxical increase in cell density (cell number per area). When considered as groups, based on the presence or not of HS, some patterns did emerge. Several observations pointed to regional pathology preferentially involving the MD in UHS, with evidence of lateralization to the side of sclerosis. We demonstrated significantly lower mean neuronal density ipsilateral to side of sclerosis in UHS cases in the MD compared to the contralateral side. Within individual HS cases, greater GFAP, lower synaptophysin and neuronal density between sides were more consistently seen in the MD than other nuclei, lateralizing to the side of sclerosis. Such comparisons within individual cases may prove a more reliable measurement than comparison between postmortem groups, as technical variations in staining dependent on tissue quality and fixation times are circumvented; in addition, the anatomic coronal level through thalamic subnuclei are more exactly matched when sampling tissue blocks. These findings could support the notion that pathology in the MD is more consistently present in HS associated with epilepsy.

The MD receives incoming networks from the amygdala, entorhinal cortex, and temporal pole (Nieuwenhuys et al., 2008a) and is connected to the prefrontal cortex; it has physiologic roles in cognition and working memory (Watanabe & Funahashi, 2012). There is experimental evidence supporting a role for the MD in epilepsy.

Cassidy and Gale reported that pretreatment of the MD with inhibitory agents protected against the development of seizures (Cassidy & Gale, 1998). Bertram also demonstrated seizure activity within the midline thalamus occurring simultaneously with the onset of seizures evoked by hippocampal stimulation in rats (Bertram et al., 2001), as well as induction of hippocampal seizures through stimulation of the MD (Bertram et al., 2008). This was found to be specific to the MD, implicating this thalamic region in the initial stages of limbic seizures. Furthermore, the seizures stimulated from MD were found to quickly generalize to surrounding areas, supporting a role for the MD in seizure spread (Bertram et al., 2008). Histologic studies in experimental models of MTLE have also demonstrated significant neuronal loss in the MD compared to controls (Bertram et al., 2001). In surgical series of patients with TLE, examination of the thalamus is clearly not possible and human histologic studies are dependent on postmortem series. In the only previous postmortem study by Margerison & Corsellis (1966) in 55 patients with TLE, thalamic “scarring” was seen in 25% of cases, more often in association with HS. In this study, they noted that the severity and distribution of damage varied, but with no predilection for a particular nuclear group. However, in their study, quantitative immunohistochemistry was not carried out as in the present study, which may have the power to detect and evaluate more subtle pathology.

Stereoelectroencephalography studies in human TLE have shown synchrony between the thalamus and cortex in patients with TLE (Guye et al., 2006); it has been suggested that the thalamus could act as a seizure amplifier and synchronizer of ictal activity (Guye et al., 2006). Magnetic resonance imaging (MRI) studies have greatly advanced our understanding of associated alterations of the thalamus in TLE. Using manual segmentation methods (Moran et al., 2001; Natsume et al., 2003), voxel based morphometry (VBM) (Bonilha et al., 2004), and diffusion tensor imaging (DTI) (Kim et al., 2010) volume reductions in the thalamus have been shown ipsilateral to the side of seizure onset (DeCarli et al., 1998; Dreifuss et al., 2001; Moran et al., 2001; Natsume et al., 2003; Bonilha et al., 2004; Kim et al., 2010; Mueller et al., 2010; Bonilha et al., 2010b; Alhusaini et al., 2012) associated both with (Alhusaini et al., 2012; Kim et al., 2010; Mueller et al., 2010) and without HS (Natsume et al., 2003). Some MR studies support greater involvement of the anterior thalamus (Bonilha et al., 2005; Mueller et al., 2010), and there are contradictory studies suggesting relative differences in the extent of contralateral thalamic atrophy associated with right-versus left-sided HS (Morgan et al., 2012; Pail et al., 2010). We failed to detect any differences in the lateralization of thalamic pathology between right-or left-sided CHS cases. A more recent MRI study, has mapped and localized thalamic atrophy to the ipsilateral medial thalamic surface (Bernhardt et al., 2012), which is comparable to our identification of pathology preferentially localizing to the MD nucleus. Positron emission tomography (PET) studies have also localized hypometabolism in the ipsilateral MD subnuclei in patients with TLE (Juhasz et al., 1999).

Clinical and imaging studies have also been evaluated in an attempt to gain further insight of the cause and progression of thalamic pathology. Natsume showed a negative correlation between thalamic volume and duration of TLE in 40 patients with TLE (Natsume et al., 2003). Duration of epilepsy was also shown to correlate with progressive thalamic changes observed in patients with HS using DTI (Keller et al., 2012). Not all studies, however, have shown a relationship between loss of thalamic volume and duration of epilepsy (Alhusaini et al., 2012). The question of progressive changes becomes more difficult to address in postmortem tissues from patients mainly at the end-stage of disease, but we did identify an association between the extent of gliosis in the VL and AV and total duration of epilepsy. A similar association was not seen for age at death, suggesting that this may represent a seizure-related effect.

A further proposal, that thalamic pathology represents transsynaptic degeneration following secondary deafferentation in hippocampal networks, was supported in one recent study combining volumetric MRI study with tractography, suggesting a relationship between alterations observed with these methods (Bonilha et al., 2010b). The evidence for loss of thalamic volume associated with HS in patients with mild (drug-sensitive) TLE (Labate et al., 2008) also supports this hypothesis of atrophy secondary to degeneration in the network rather than a direct seizure effect. As the main output pathway of the hippocampus, via the fornix and medial mamillary nucleus, is to the AV, we might anticipate greater atrophy in this nucleus. Indeed, stimulation of the AV has recently been trialed as an effective treatment in refractory epilepsy of temporal origin (Fisher et al., 2010), with these direct connections between the anterior nucleus and the hippocampus implicated in this therapeutic effect (Fridley et al., 2012). However, in our small series we failed to confirm significant pathology preferentially involving the AV, either in single cases or in groups of HS cases. Finally, MRI studies have shown smaller thalamic volumes with a history of febrile seizures as a potential precipitating insult for thalamic pathology (Natsume et al., 2003); it was not possible to investigate the contribution of initial injury to thalamic pathology in the present clinical series because of the lack of sufficient early historic data in many cases.

There are several limitations in the present study. One of the main ones being that this postmortem group represents a more heterogenous cohort than surgical epilepsy series, particularly in terms of the epilepsy syndrome in that not all patients have MTLE, which may partly explain the lack of consistent thalamic pathology observed. Tissue processing and differences in fixation times can influence the intensity of immunostaining. We attempted to overcome this problem by comparing the left and right sides within individual cases in addition to mean values between groups. Although we aimed to exclude confounding pathologies, we cannot exclude, particularly in older patients, the contribution of subclinical cerebrovascular disease to thalamic gliosis and neuronal loss. Not all thalamic subnuclei were represented in each case, and the thalamic coronal levels at which subnuclei were sampled, varied between cases, which could influence data. In addition, we did not subdivide the nuclei further, which may reduce the sensitivity of detecting focal pathology. A further prospective PM study with sampling through the entire thalamus, utilizing three-dimensional stereologic analysis, may be required to further study the MD in detail.

In summary, our PM study demonstrates that stereotypical pathologic changes, as seen in HS, are not consistently or clearly defined in the thalamus. We did identify a predilection for neuronal and synaptic loss and gliosis in the MD, in keeping with previous clinical and experimental data, supporting the potential role of the MD in epilepsy. This is an area that warrants further detailed investigation.
